# Auto-segmentation and time-dependent systematic analysis of mesoscale cellular structure in *β*-cells during insulin secretion

**DOI:** 10.1371/journal.pone.0265567

**Published:** 2022-03-24

**Authors:** Angdi Li, Xiangyi Zhang, Jitin Singla, Kate White, Valentina Loconte, Chuanyang Hu, Chuyu Zhang, Shuailin Li, Weimin Li, John Paul Francis, Chenxi Wang, Andrej Sali, Liping Sun, Xuming He, Raymond C. Stevens

**Affiliations:** 1 iHuman Institute, ShanghaiTech University, Shanghai, China; 2 School of Life Science and Technology, ShanghaiTech University, Shanghai, China; 3 University of Chinese Academy of Sciences, Beijing, China; 4 School of Information Science and Technology, ShanghaiTech University, Shanghai, China; 5 Shanghai Engineering Research Center of Intelligent Vision and Imaging, Shanghai, China; 6 Department of Biological Sciences, Bridge Institute, University of Southern California, Los Angeles, CA, United States of America; 7 Department of Computer Science, Bridge Institute, USC Michelson Center for Convergent Bioscience, University of Southern California, Los Angeles, CA, United States of America; 8 California Institute for Quantitative Biosciences, Department of Bioengineering and Therapeutic Sciences, Department of Pharmaceutical Chemistry, University of California, San Francisco, San Francisco, CA, United States of America; J. Heyrovsky Institute of Physical Chemistry, CZECH REPUBLIC

## Abstract

The mesoscale description of the subcellular organization informs about cellular mechanisms in disease state. However, applications of soft X-ray tomography (SXT), an important approach for characterizing organelle organization, are limited by labor-intensive manual segmentation. Here we report a pipeline for automated segmentation and systematic analysis of SXT tomograms. Our approach combines semantic and first-applied instance segmentation to produce separate organelle masks with high Dice and Recall indexes, followed by analysis of organelle localization based on the radial distribution function. We demonstrated this technique by investigating the organization of INS-1E pancreatic *β*-cell organization under different treatments at multiple time points. Consistent with a previous analysis of a similar dataset, our results revealed the impact of glucose stimulation on the localization and molecular density of insulin vesicles and mitochondria. This pipeline can be extended to SXT tomograms of any cell type to shed light on the subcellular rearrangements under different drug treatments.

## Introduction

The mesoscale describes a spatial scale ranging from protein complexes (50–100 nm) to whole cells (∼ 10 *μ*m), spanning a range in which all organelle rearrangements and cellular architectures can be observed. Studies at the mesoscale bridge living processes that occur at the cellular-scale to atomic interactions on the protein-scale [1]. This includes changes in volumes, molecular composition, and distributions of organelles as well as inter-organelle interactions that achieve various cellular functions. A comparison of mesoscale organization between healthy and disease states will shed light on the cellular mechanisms of disease, like, for example, the impaired *β*-cell function of type 2 diabetes about which many aspects of subcellular reorganization remain unknown [2].

Soft X-ray tomography (SXT) is an important technique for revealing the mesoscale rearrangements of organelles of single-cells in a near-native state [3, 4]. SXT can reach resolutions of tens of nanometers (∼ 25 nm) [5], where organelles like mitochondria and insulin vesicles can be clearly visualized. Because SXT allows for rapid data collection and does not require cell sectioning, numerous conditions can be tested such as different drug conditions and time points. Several single-cell studies have been made via SXT techniques [6, 7]. However, despite the relative rapidity of collecting SXT tomograms (i.e. on the order of minutes), the manual segmentation of a single cell, which takes hours to several days, represents a real efficiency bottleneck. This time-consuming segmentation step severely restricts downstream analysis, especially for studies comparing multiple drug stimulations. Moreover, manual identification of organelles also introduces bias to the segmentation masks, potentially introducing error in the analysis.

Auto-segmentation is advantageous compared to manual segmentation in two aspects: 1) eliminate the manual biases, 2) produce larger segmented datasets [8–10]. It has been widely applied to various medical and biological images aiming to classify image pixels with semantic labels (semantic segmentation) or further separate object instances in the same semantic class (instance segmentation). For semantic segmentation, several well-known architectures have been proposed over past years, including fully convolutional networks (FCN) [11] and u-shaped architecture network (U-Net) [12]. As to instance segmentation, many convolutional neuron network (CNN) based architectures have been developed recently including region based convolutional neuron network (R-CNN), Fast R-CNN [13], Faster R-CNN [14], and Mask R-CNN [15]. These methods are mainly focused on segmenting magnetic resonance images [16–20], computed tomography data [21–25], and fluorescent microscopy images [26–29]. To the best of our knowledge, little work has been done for SXT tomograms using semantic segmentation [30, 31], none using instance segmentation.

With the segmented masks in hand, SXT tomograms have been analyzed in various ways to study organelle arrangement in the mesoscales. For example, *Uchida et al*. [32] focused on the morphological variances like nucleus and mitochondria volume ratio under antifungal peptoids treatment in *C.albicans* cells. *Ma et al*. [33] used SXT to simulate the intracellular molecular dynamics and transmissions with organelles as barriers. In addition, *White et al*. [3] mainly investigated the organelle interactions and related localization in *β*-cells. However, systematic analysis containing both localization and morphologies variances to study single-cell SXT has not been proposed yet.

To address which, we present a two-branch auto-segmentation method combining semantic and instance segmentation for the first time, followed by a systematic analysis pipeline to quantify a series of time-resolved soft X-ray tomograms. To demonstrate the value of this novel segmentation approach, we apply it to the investigation of subcellular rearrangements of pancreatic *β*-cells, validate it using morphological analysis of 40 manually segmented SXT tomograms reported by *White et al*. [3], and extend it to 132 SXT tomograms. In these cells, insulin is transported in vesicles via microtubule networks to the plasma membrane [34]. Insulin vesicles are stored in reserve pools and readily releasable pools (RRP) [35]. An increase in extracellular glucose concentration leads to the fusion of insulin vesicles with the plasma membrane, releasing the insulin into the bloodstream. Currently, a number of potential drugs have been discovered for treating diabetes, for instance, exendin-4 (Ex-4) [36] and tifenazoxide (NN414) [37]. However, the global effects of these drugs on mesoscale architecture are still uncertain. Tomograms for this application are collected on INS-1E cells (a rat insulinoma cell line) [38], treated with various combinations of 25 mM glucose, 10 nM Exendin-4 (or Ex-4, a glucagon-like peptide-1 receptor agonist that enhances glucose stimulated insulin secretion), and 30 *μ*M NN414 treatment (a selective SUR1/Kir6.2 potassium channel opener that inhibits insulin secretion). Additionally, we have a control condition (no external stimuli) and 50 mM KCl treatment that induces insulin secretion by a mechanism independent of glucose metabolism.

## Materials and methods

### Two-branch segmentation

**Fig 1:**
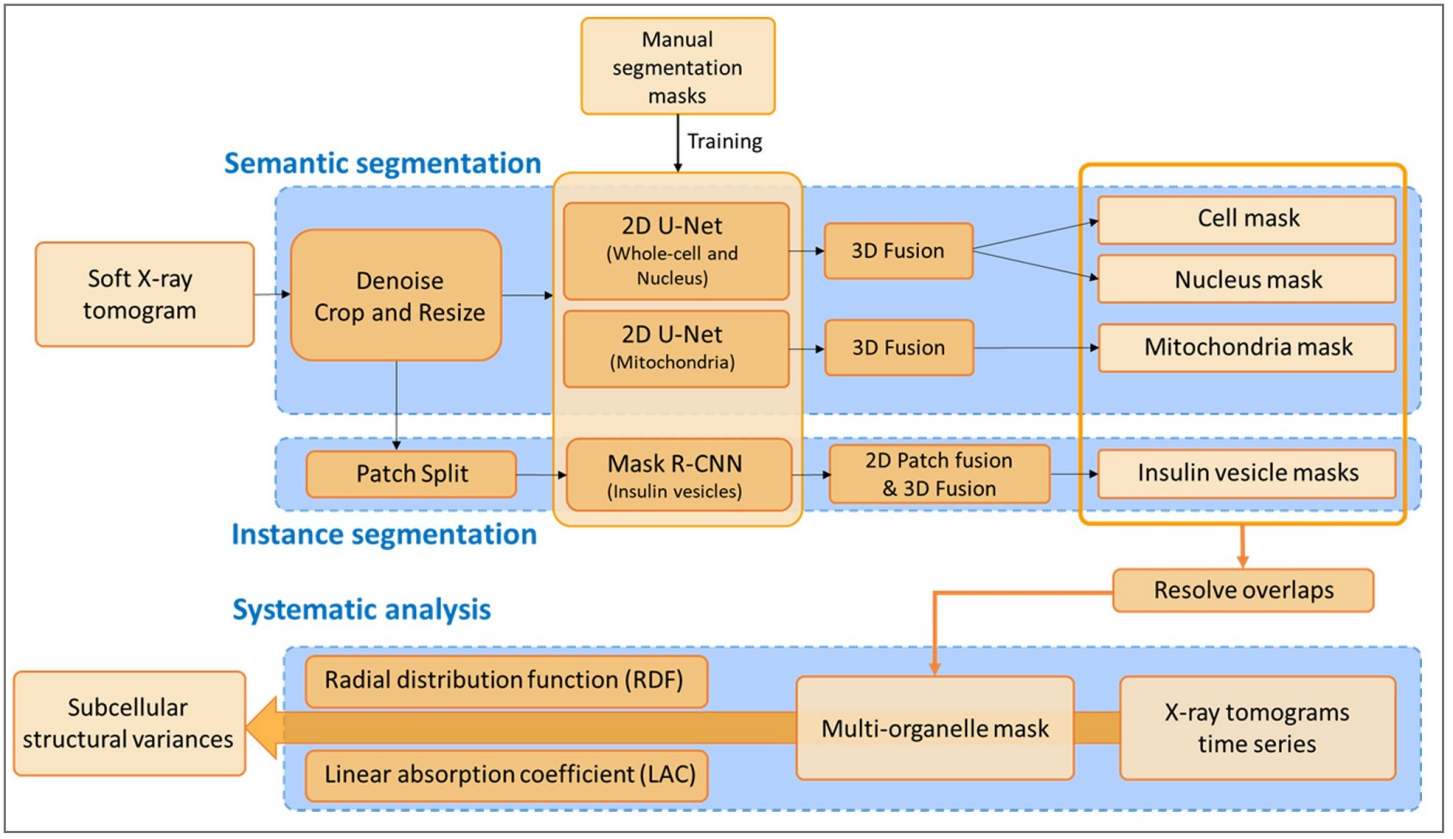
Complete workflow of the segmentation and analysis of time-resolved soft X-ray tomograms (SXT). Blue boxes highlight the three different frameworks: semantic segmentation, instance segmentation, and systematic analysis. Dark orange boxes show the steps of each framework; light orange boxes represent data at different stages; black arrows indicate the direction of data processing; and the thick orange arrow represents multiple and simultaneous tomograms processing. The predicted 2D labels of ‘cell’, ‘nucleus’, ‘mitochondria’ and individual ‘insulin vesicle’ masks were combined to generate 3D organelle masks. Then these 3D masks were merged together based on the priority (Method: Systematic analysis) to get the multi-organelle mask.

Our method contains a deep segmentation network with two branches. First, a semantic segmentation branch generates category-level masks for the mitochondria, nucleus, and cell. Second, an instance segmentation branch produces individual masks for insulin vesicles since separate instances of vesicles are required for the systematic analysis. Then we merge the organelle masks generated from the two branches to construct a final segmentation of the entire cell using a priority-based fusion mechanism. The detailed workflow of the whole segmentation process is shown in Fig 1. The segmentation and analysis codes used are available at https://github.com/SaliLab-SH/Cell-Segmentation.

### Semantic segmentation

The morphologies of cell and nucleus were comparable: large volume (cell and nucleus occupy approx. 16% and 5% of one tomogram, respectively) and aggregating in one region of the tomogram. However, the morphology of mitochondria was quite different from cell and nucleus: small volume (<2% of one tomogram) and spreading over the cytoplasm. Such morphological differences led to extremely time-consuming convergence when training a single multi-class U-net for cell, nucleus, and mitochondria, raising difficulty in parameter adjustment during model optimization. **Thus, we trained two separate U-Net networks, one for the nucleus and cell, and the second for mitochondria**. We trained two separate U-Net networks, one for the nucleus and cell and the second one for mitochondria. U-Nets were trained on 2D slices obtained from 3D tomograms. Specifically, after preprocessing, each tomogram was sliced into 280 2D slices along the axial (*X*) direction for the cell and nucleus segmentation, whereas we used 2D slices from all three axes for the mitochondrial segmentation, including 320 along the axial (*X*) and coronal (*Y*) directions and 420 along the sagittal (*Z*) direction (1060 2D slices in total for each 3D tomogram).

#### Pre-processing

The first step in segmentation was preprocessing of the tomograms, which included noise removal and resizing.

Noise Removal: In order to remove noise from the images, we adopted the Noise2Void method proposed by [39]. Noise2Void uses convolution neural networks to learn about the properties of images and how to reduce noise without supervision. It aims to produce a regularized denoised image as output. We used the package in ImageJ to process the 3D X-ray tomograms. The Noise2Void can be downloaded at https://imagej.net/N2V.

Crop and Resize: Since the tomograms were collected by placing cells in the glass capillary, each slice was cropped by the capillary lines in the tomogram (S1B Fig). Cropping based on capillary walls creates different sized 3D images, so we resized the cropped images to a standard width, height, and depth of 280, 480, and 320 pixels, respectively.

#### Segmentation using U-Net

In this work, we adopted an effective classical deep-learning model U-Net [12] for semantic segmentation. The main architectural design of the method is the combination of layer downsampling and upsampling, with skip connection between them as illustrated in S2 Fig. The first part is the contraction path (left side), also called the encoder, which is used to capture image context. The second part is the expanding path (right side), which is used to enable precise localization by deconvolution. Thus, the two sides combined form a U-shaped architecture that allows the network to propagate information collected from a larger receive field of images. To achieve more precise localization using multiple features, at every step U-Net uses skip connections that concatenate the output of the deconvolution layers with the feature maps from the encoder at the same level.

For our task, each U-Net uses an encoder that sequentially applies a convolutional block of two 3*3 convolution layers followed by a rectified linear unit (ReLU) and a 2*2 max-pooling with stride 2. At each decoding step, it adopts a deconvolutional layer that first interpolates the feature from its previous layer, concatenates with the corresponding skipped feature from the contracting path, and then goes through two 3*3 convolutions, each followed by a ReLU. Given the upsampled feature, we finally applied a 1*1 convolution layer on each feature element to get the predicted segmentation mask. To build the whole layer-by-layer process, we optimize the parameters of each layer, denoted as *W*, by minimizing the training objective: cross-entropy loss *L*_*ce*_ between the predicted mask $\hat{y_{i}}$ and ground truth manual segmentation *y*_*i*_ for each category *i*, using stochastic gradient descent.

$$\begin{array}{c} L_{ce}=-\sum_{i}y_{i}log\left(\hat{y_{i}}\right) \end{array}$$ (1)

#### 3D Fusion post-processing

Since semantic segmentation was performed on 2D images, we merged the 2D labels into 3D organelle masks. To improve the performance, we predicted binary 2D labels from all three axes for cell, nucleus and mitochondria labels (S3 Fig). To generate the 3D masks of organelle, we developed a multi-view fusion algorithm that utilizes the complementary information to fix the uncertainty from the isolated prediction of three views. If the raw 3D volume data is represented as $V\in R^{3}$, our goal was to assign the label to each voxel *v*_*i*_ ∈ *V*. First, we construct 3D label masks by simply merging 2D labels in each view, i.e. *V*^*X*^,*V*^*Y*^, and *V*^*Z*^. Each voxel *v*_*i*_ ∈ *V* in the raw tomogram has three predictions available from each view, corresponding to $v_{i}^{X}\in V^{X}$, $v_{i}^{y}\in V^{Y}$ and $v_{i}^{Z}\in V^{Z}$. Then, the final binary fused prediction is generated using the voting strategy as follows: *v*_*i*_ = 1 if $v_{i}^{X}+v_{i}^{Y}+v_{i}^{Z}>=2$, else *v*_*i*_ = 0. Improvements in the segmentation accuracy is seen for the semantic masks of cell, nucleus, and mitochondria after the application of 3D fusion post-processing (S1 Table).

On a 4 TITAN Xp GPU setup it took 5–6 hours of training for the semantic segmentation on each U-Net (18 tomograms) and around 10 hours of predicting on each U-Net on a total of 132 tomograms.

### Instance segmentation

The instance segmentation was performed for insulin vesicles using the Mask R-CNN network [15]. In addition to the noise removal, cropping, and split preprocessing steps mentioned in the semantic segmentation section, an additional preprocessing step, called Patch Split, was also performed for the segmentation of insulin vesicles.

#### Pre-processing

Patch Split: Detecting the object granule (insulin vesicle) is challenging since the vesicles are small and tend to vanish in the max-pooling layer. Therefore, we decided to crop the raw 2D image (280 × 480 pixels) into several image patches before feeding them into the network. Each patch was about 150 × 50 pixels and overlapped about 30% with each other.

#### Segmentation using Mask R-CNN

Mask R-CNN [15] is one of the state-of-the-art models for instance segmentation, developed on top of Faster R-CNN [14], which adopts the two-stage procedure shown in S2B Fig. The first stage generates the candidate bounding boxes for each object region. Specifically, the backbone network exploits a Deep Residual Network (ResNet) [40] with a Feature Pyramid Network (FPN) [41]. It consists of bottom-up and top-down pathways as well as lateral connections. The bottom-up pathway extracts features from raw images and the top-down pathway generates a feature pyramid map similar in size to the bottom-up pathway. Lateral connections merge feature maps of the same spatial size from the corresponding levels in the two pathways. This operation captures strong representative image features from multi-scale resolution images. Then, a lightweight neural network called Region Proposal Network (RPN) [14] identifies regions in the feature map that may contain objects, also called Regions of Interest (RoI).

In the second stage, the network predicts the classification output and refines the bounding box localization, as well as generating a segmentation mask at the pixel level. First, each RoI is pooled into a fixed-size feature map and then fed into two branches of predictions. The first branch predicts a class label and the bounding-box regression offsets for each RoI using fully connected layers (FCs). The second branch generates mask logits output per pixel for each granule at the pixel level.

#### Post-processing

Restoring the prediction to original image size: Since our prediction is based on image patches, these needed to be fused together to restore the original image size. In the overlapping area between two patches, the prediction with a higher score was retained.

3D Fusion: 3D fusion means we combine the instance segmentation from current 2D images to 3D instance. To achieve 3D fusion, we set an Intersection-over-Union (IoU) as a metric for the predicted segmentation mask on each 2D slice to determine if 2D instances belong to the same 3D instance. The instance IoU is calculated as the ratio of the overlapping area of the predicted masks to the total combined area from the adjacent slices. If the IoU is greater than the threshold of 0.5, the 2D masks are kept as the same instance mask, otherwise the masks are regarded as isolated instances.

On a 4 TITAN Xp GPU setup it took 15–16 hours of training for the instance segmentation with Mask R-CNN (18 tomograms) and around 20 hours of predicting on a total of 132 tomograms.

### Systematic analysis

#### Linear absorption coefficient (LAC)

LAC is a linear function of thickness and chemical composition [42]. It reflects the absorption of X-ray samples as well as the molecular density with small regions. Regions with varying LAC were used to identify organelles in SXT data.

#### Fusion of different organelle labels

We set the priority for each label mask as: insulin vesicle mask >nucleus mask >mitochondria mask >cell mask. Masks with high priority replaced masks with low priority where they overlapped.

#### Metrics to evaluate the segmentation model

The Dice Coefficient is a commonly used metrics in semantic segmentation, which is calculated as follows:

$$\begin{array}{c} dice=\frac{2*|\hat{y_{i}}\cap y_{i}|}{|\hat{y_{i}}\left|+\right|y_{i}|} \end{array}$$ (2)

Here $|\hat{y_{i}}\cap y_{i}|$ denotes the overlapping area between the predicted masks, $\hat{y_{i}}$, and the ground truth manual segmentation, *y*_*i*_.

Recall, which measures how many of the actual positives our model captures, is defined as the number of true positives, *T*_*p*_, over the number of *T*_*p*_ plus the number of false negatives, *F*_*n*_. We define the distance function *d* as follows to measure the *T*_*p*_.

$$\begin{array}{c} d=\frac{d_{u}}{r_{\hat{y_{i}}}+r_{y_{i}}} \end{array}$$ (3)

where *d*_*u*_ is the Euclidean distance between the center of $\hat{y_{i}}$ and *y*_*i*_. $r_{\hat{y_{i}}}$ and $r_{y_{i}}$ are are the radii of the predicted mask, $\hat{y_{i}}$, and manual segmentation, *y*_*i*_, respectively. If *d* < 1, we treat it as *T*_*p*_.

#### Radial distribution function (RDF)

RDF was used to calculate the probability of organelle *B*’s appearance at a distance *r* from organelle *A*. [43] The probability *g*(*r*) is defined as:

$$\begin{array}{c} g_{AB}\left(r\right)=\frac{\left\langle \rho_{B}\left(r\right)\right\rangle }{{\left\langle \rho_{B}\right\rangle }_{local}}=\frac{1}{{\left\langle \rho_{B}\right\rangle }_{local}}\frac{1}{N_{A}}\sum_{i\in A}^{N_{A}}\sum_{j\in B}^{N_{B}}\frac{\delta (r_{ij}-r)}{4\pi r^{2}} \end{array}$$ (4)

where *ρ*_*B*_(*r*) is the average density of *B* at a distance *r* around *A*. 〈*ρ*_*B*_〉_*local*_ is the average density of *B* for all spheres around *A*. *δ*(*r*_*ij*_ − *r*) is the dirac delta function. In our calculation, insulin vesicle RDF is calculated on numbers since we have the instance segmentation on insulin vesicle masks. Mitochondria RDF is calculated on voxel distribution. Here we used RDF to divide cytosol into 8 shells, as shown in S2C Fig.

Insulin vesicle RDF are calculated by computing the center of each insulin vesicle instances distribution. Mitochondria RDF are calculated by computing the mitochondria voxels distribution. Insulin vesicle-mitochondria RDF are calculated by computing the center of each contact. A single contact is defined as a region that insulin vesicle voxels close to mitochondria surface with distance less than 1.5 voxels.

## Results

### Auto-segmentation of 3D Soft X-ray tomograms

#### Auto-segmentation workflow

The first step in our method is the segmentation of single-cell tomograms (S1 Fig) to generate organelle masks for analysis using our auto-segmentation method, which combines semantic and instance segmentation frameworks(see Methods section for more detail). We used the U-Net [12] framework (S2A Fig) to train and segment the ‘cell’, ‘nucleus’, and ‘mitochondria’ labels and to obtain semantic segmentation masks. For ‘insulin vesicle’ labels we trained the Mask R-CNN framework [15] (S2B Fig) to obtain instance segmentation masks. In total, we used 132 tomograms in this study, out of which manual segmentation was available for only 24 tomograms. The 24 tomograms are randomly divided into three partitions: 18 for training, 3 for validation, and 3 for testing. The accuracy of the auto-segmentation was assessed using the test dataset and quantified using the Dice coefficient for U-Net [12] and the Recall for Mask R-CNN [15]. After segmentation evaluation, we applied the trained networks to the complete set of 132 single-cell tomograms and obtained 3D masks for cells and organelles including the nucleus, mitochondria, and insulin vesicles. The complete segmentation workflow is shown in Fig 1 with details provided in the Methods section.

#### Auto-segmentation accuracy

**Fig 2:**
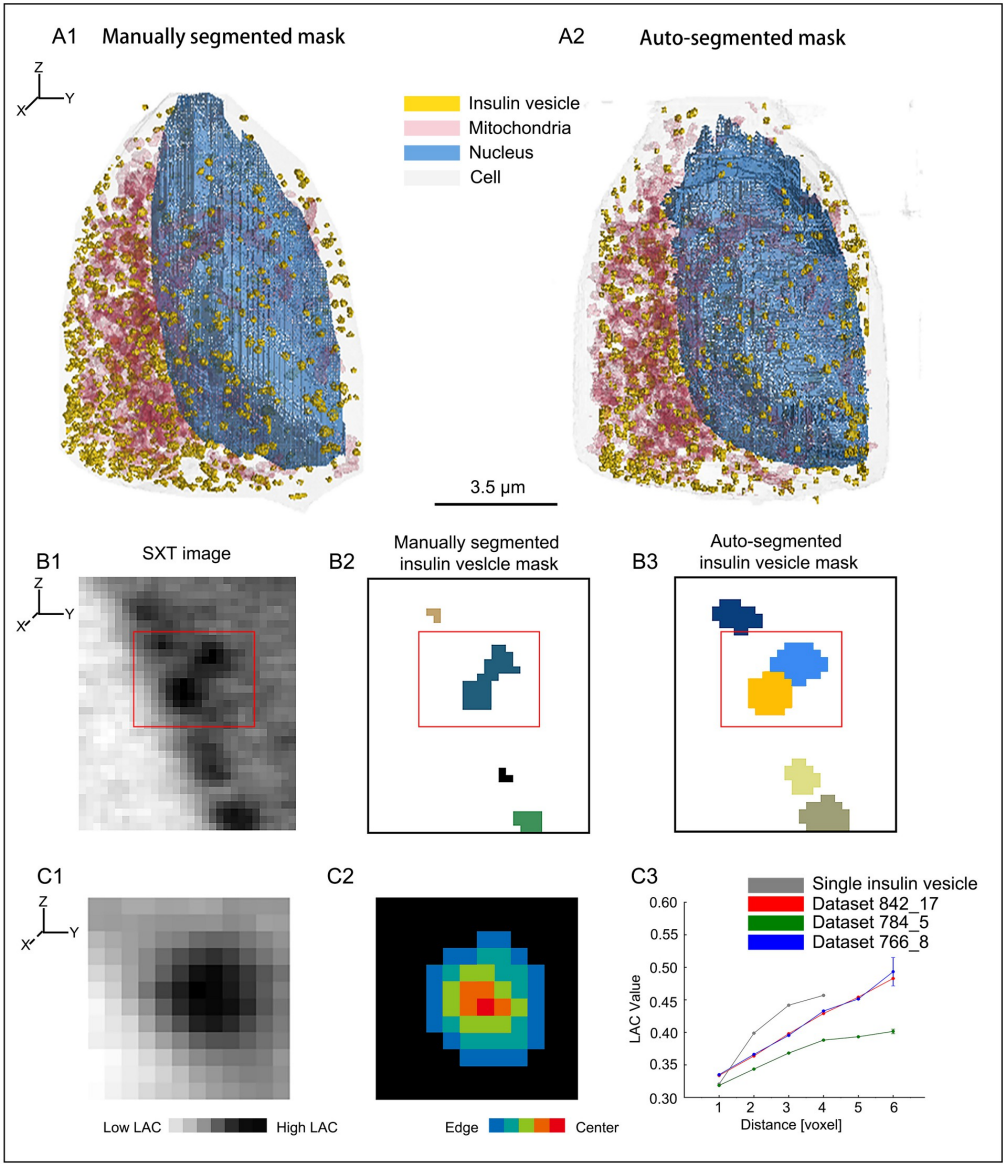
Example of auto-segmentation and performance on the test dataset. (A1-A2) 3D visualization to represent labels for Cell ID 766_8: (A1) manually segmented labels and (A2) auto-segmented labels. (B1-B3) Cropped 2D orthoslice of raw soft X-ray tomogram for Cell ID 842_17. Red box shows two vesicles near the plasma membrane (B1). Manually segmented mask where two vesicles are merged into one (B2). Auto-segmented mask showing correct prediction based on instance segmentation (B3). Each color represents a single instance. (C1-C3) 2D orthoslice of single insulin vesicle instance from soft X-ray tomogram of Cell ID 842_17. The voxel with the highest linear absorption coefficient (LAC) value was assigned as the center of the vesicle. LAC map of the vesicle and surrounding pixel (C1). Distance map from vesicle boundary (C2). Average LAC vs. distance of the vesicle instance from C2 shown as “Single insulin vesicle” (C3). Average LAC distribution for 3 test cells is also plotted in C3.

To measure the accuracy of our segmentation results, we first computed the Dice coefficient and Recall for measuring the accuracy of the U-Net and Mask R-CNN networks for each of the three training datasets (Table 1), then we investigate the various features from our segmented masks based on linear absorption coefficient (LAC) and radial distribution function (RDF) (see Materials and methods section: Systematic analysis: Linear absorption coefficient(LAC), Radial distribution function (RDF)). The auto-segmentation results for Cell ID 766_8 are reported in Fig 2A and 2B together with the manually segmented masks.

**Table 1:** Auto-segmentation accuracy: Dice, Recall, *AP*_50_, and insulin vesicle numbers on test datasets compared to manual segmentation [3].

Dataset	U-Net			Mask R-CNN			Number of Insulin vesicles	
	Dice coefficient(%)			Recall (%)	Dice coefficient*(%)	*AP*_50_ (%)	Manual segmentation	Auto-segmentation
	Cell	Nucleus	Mitochondria	Insulin vesicle	Insulin vesicle	Insulin vesicle		
766_8	93.54	93.92	70.34	84.20	33.27	64.07	787	591
784_5	89.41	91.82	67.29	87.56	9.95	26.15	303	862
842_17	91.85	89.49	67.40	95.51	52.59	32.28	340	643
Mean	91.60	91.74	68.34	89.09	31.94	40.83	476.67	698.67

*Dice coefficient for mask R-CNN is computed using insulin vesicle semantic masks, converted from the corresponding instance masks.

The 2D mask predictions of cell and nucleus labels were quite accurate, with the mean Dice coefficient >91%, confirming high quality auto-segmentation. However, predictions made for mitochondria showed that the Dice coefficient only reached ∼70% accuracy. To investigate the relatively low accuracy of mitochondria masks, we first computed the contrast ratio, i.e., the normalized intensity value of the mitochondria mask in 2D slices divided by the surrounding cytoplasmic pixels in the manual segmentations (Table 2). The resulting low contrast ratio (approx. 1.1) revealed that mitochondria were only marginally distinct from the cytoplasmic surroundings, consequently explaining the lower accuracy of the mitochondria label prediction.

**Table 2:** Linear absorption coefficient (LAC) value and normalized intensity of insulin vesicle and mitochondria masks in the three test datasets from the manual segmentation results.

Test Dataset	Insulin vesicle mask	Mitochondria mask	Insulin vesicle surrounding voxels	Mitochondria surrounding voxels	Insulin vesicle mask contrast ratio	Mitochondria mask contrast ratio
	Normalized intensity	Normalized intensity	Normalized intensity	Normalized intensity	Insulin vesicle normalized intensity / insulin vesicle surrounding voxels normalized intensity	Mitochondria mask normalized intensity / mitochondria surrounding voxels normalized intensity
766_8	0.3573	0.2847	0.2976	0.2562	1.2006	1.1113
784_5	0.4561	0.4227	0.3768	0.3725	1.2104	1.1347
842_17	0.5227	0.3898	0.4291	0.3580	1.2180	1.0886
Mean	0.4453	0.3657	0.3678	0.3289	1.2097	1.1115

Normalized intensity of a pixel was calculated as pixel intensity divided by the maximum intensity of that 2D orthoslice.

For the insulin vesicle instance segmentation results, the mean Recall was high with the value of ∼89%, indicating high prediction accuracy. However, the mean Dice coefficient and the mean *AP*_50_ (average precision with an IoU threshold of 50%) only reached 32% and 41%, respectively. Note that the Dice coefficient was calculated using semantic masks converted from the corresponding instance masks, whereas *AP*_50_ was computed using instance masks directly. Noteworthy, the contrast ratio of insulin vesicles in the tomogram of cell ID 766_8 was quite low (Table 3)), raising difficulties in both manual segmentation and auto-segmentation, indicated by low Dice coefficient and *AP*_50_. Despite the low quality of this tomogram (will be discussed with more details below, Table 3, S4 Fig), we still achieved a Recall of 84%, confirming the high accuracy of auto-segmentation in reproducing manual segmentation results. For the tomograms of cell ID 784_5 and 842_17, manual segmentation results were nicely reproduced, indicated by the high Recall. Moreover, auto-segmentation predicted hundreds of additional insulin vesicle instances on regions that were not containing labels in manually segmented masks, indicated by low Dice coefficient and *AP*_50_. This further justified the benefit of auto-segmentation in recognizing insulin vesicle instances that could not be recognized in manual segmentation (S5 Fig).

**Table 3:** Table 2 continuous: Linear absorption coefficient (LAC) value and normalized intensity of insulin vesicle mask in the three test datasets for those missed insulin vesicles masks.

Dataset (missed insulin vesicle masks)	Insulin vesicle mask	Insulin vesicle surrounding voxels	Insulin vesicle mask normalized intensity contrast ratio
	Normalized intensity	Normalized intensity	(Insulin vesicle intensity / insulin vesicle surrounding voxels intensity) (%)
766_8	0.2980	0.2675	1.1141
784_5	0.4083	0.3514	1.1618
842_17	0.4172	0.3565	1.1704
Mean	0.3745	0.3251	1.1488

Missed insulin vesicles were calculated by matching manual segmented instances to auto-segmented instances, and unmatched instances from the manual segmented results we classified as the missed insulin vesicles.

To investigate the relative low accuracy of mitochondria masks, we computed the contrast ratio and compared it with insulin vesicle, i.e. the normalized intensity value of the organelle mask in 2D slices divided by the surrounding cytoplasmic pixels in the manual segmentations. We observed that the contrast ratio of mitochondria was lower than that of the insulin vesicles (Tables 2 and 3), revealing that mitochondria are less distinct from the cytoplasmic surroundings, consequently explaining the lower accuracy of the mitochondria label prediction.

The instance segmentation of insulin vesicles using Mask R-CNN also provided the number of insulin vesicle for each cell (Table 1). We noticed in two of the three test datasets and on average that the insulin vesicle numbers from the auto-segmentation results were higher than the manually segmented vesicle numbers. This was mainly due to the fact that, in the manual segmentation process, clusters of insulin vesicles were counted as one single instance, whereas the trained network was able to discriminate single instances (Fig 2B1–2B3), further justifying the benefit of using instance segmentation for insulin vesicles. Moreover, we noticed in cell ID 766_8, the number of insulin vesicles is lower from the auto-segmentation result than from the manual segmentation result. This was due to the significantly low contrast ratio of insulin vesicles in the tomogram of this cell as compared to the other two cells (Table 3). For example, one organelle labelled as insulin vesicle in manual segmentation but not in auto-segmentation (S4 Fig) has a LAC lower than the normal range of insulin vesicles (0.3–0.6) [3].

Furthermore, we investigated the quality of insulin vesicle instance segmentation (see details in the Methods section). For each individual insulin vesicle, we used the instance mask and computed the average linear absorption coefficient (LAC) value [3, 44] of voxels at different radial positions inside the vesicle (Fig 2C1–2C3). We found that the LAC value increases from the vesicle boundary to the center, confirming the dense-core feature of insulin vesicles [3, 45, 46].

### Systematic analysis of time-resolved soft X-ray tomograms

Next, we examined various features extracted from auto-segmented masks of soft X-ray tomograms collected at multiple time points as well as under different treatment conditions. We compared the variance through aspects from cell volume, nucleus volume, mitochondrial volume, mitochondrial LAC values, insulin vesicle volume and insulin vesicle LAC values under different conditions. All 132 datasets were classified into conditions as shown in S2 Table and the comparison results of these conditions were shown in S6 Fig. To validate our method, we first compared our result with *White et al*. [3] under same conditions. Then we extended our method with homemade radial distribution function (RDF) analysis to investigate the organelles rearrangement under other conditions.

#### Validation of auto-segmentation method using morphological variances

To further validate our pipeline, we investigated the subcellular variances under glucose and glucose + Ex-4 on the insulin secretion process and compared the results to those using manual segmented masks by *White et al*. [3]. Following the methodology presented in *White et al*. [3], we compared the variance on the same aspects of insulin vesicle LAC values, mitochondria LAC values and mitochondria volume (S7 Fig). We found that in both glucose and glucose + Ex-4 stimulations, especially in 5 min time point, there were significant increase in mitochondria LAC values and volumes as well as the insulin vesicle LAC values. Thus, results from our method confirmed the hypothesis that Ex-4 may affecting the trafficking system to promote insulin secretion, which was based on analysis on manual segmentation of SXT [3].

#### Functional spaces within *β*-cell architecture

The process of insulin secretion took place in two stages. The first phase happened within the first 10 min of glucose stimulation, and the second phase lasted from 10 min to four hours [47, 48]. During the first phase, insulin vesicles docked at the plasma membrane [49] are immediately secreted, decreasing the number of pre-docked vesicles. Meanwhile, other insulin vesicles in the reserve pool are transported toward the plasma membrane in preparation to be secreted [35]. During the second phase, secretion of insulin continued at a low rate with newcomer insulin vesicles contributing to the secretion [50, 51]. These new vesicles were trafficked from the area within 0.6 *μ*m of the plasma membrane [52].

**Fig 3:**
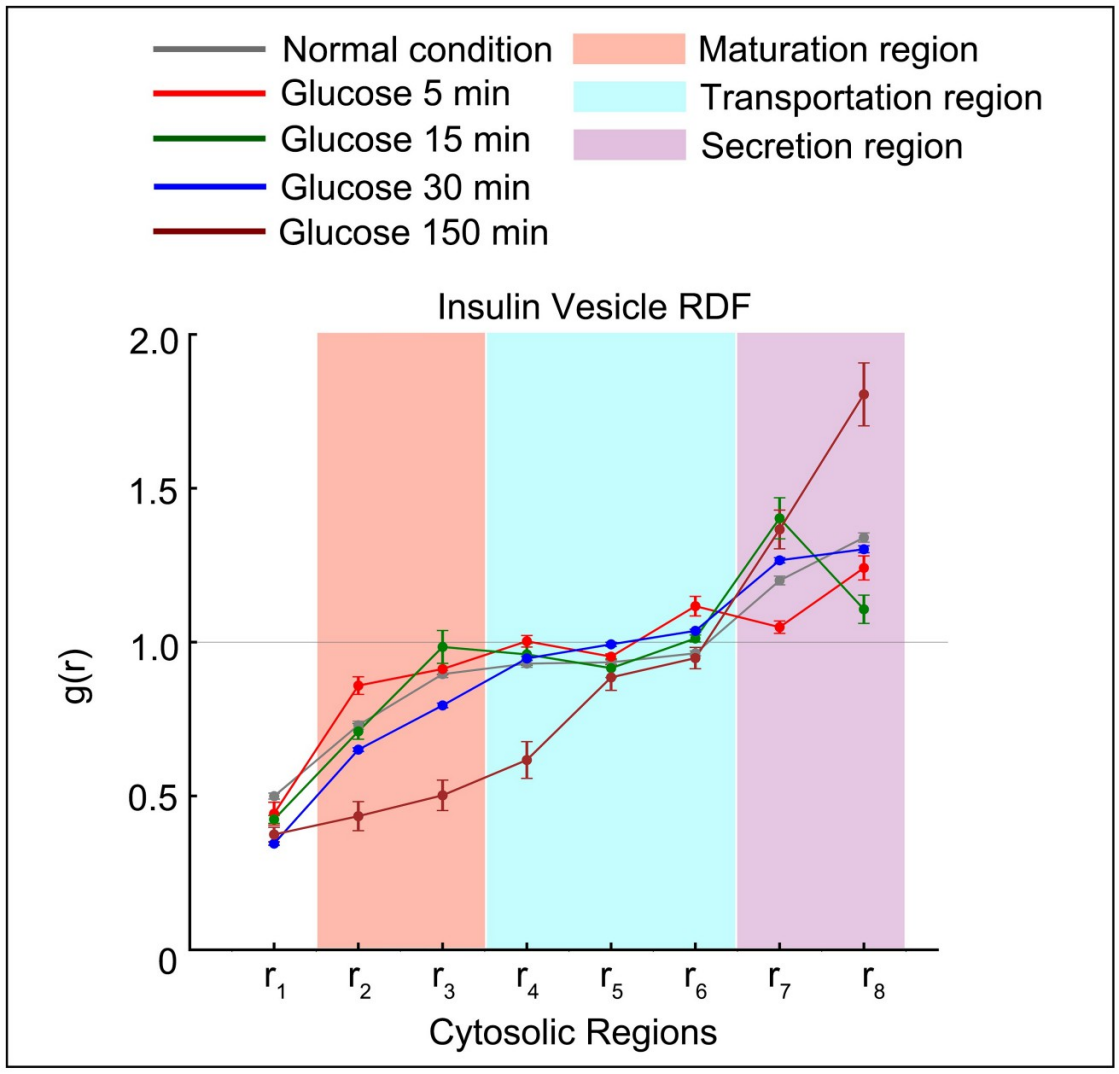
Insulin vesicle distribution and functional regions related to the insulin secretion pathway. Radial distribution function (RDF) of insulin vesicles from the nuclear membrane under the given treatment conditions. Functional spaces related to the insulin secretion pathway are shown with different background colors as indicated. The light gray horizontal line at *g*(*r*) = 1.0 shows where the probability of finding insulin vesicles in a shell is the same as random probability.

We investigated insulin vesicle distributions during the secretion by comparing the probability of insulin vesicle occurring in cytosolic regions. We compared insulin vesicle behavior after stimulation with high glucose concentration (25 mM) at four different time points (5, 15, 30, and 150 min). The condition without glucose treatment served as a baseline control (Fig 3). In order to represent insulin vesicle distribution, we applied a RDF, which calculates the probability of insulin vesicles appearing at a distance *r* from the nucleus (Methods: Radial function distribution). We divided the cytosol (non-nuclear) space into 8 concentric regions based on the distance to the nucleus membrane (S2C Fig), with region *r*_1_ closest to the nucleus and *r*_8_ closest to the plasma membrane. Firstly, we found that at 5 min (the time point that falls in the first phase of insulin secretion), the probability of insulin vesicles appearing in regions *r*_7_ and *r*_8_ was lower as compared to the control (Fig 3). This showed that insulin vesicles in the readily releasable pool (RRP), which were docked close to the plasma membrane, were released quickly as the rapid reaction to glucose stimulation. Also, at 15 min (the earliest time point in the second phase of insulin secretion), we noticed a peak in region *r*_7_ as well as a reduced probability in region *r*_8_ compared to the first phase observation, showing that newly generated insulin vesicles were being transported towards the plasma membrane while docked vesicles were still being released. Later at 30 min, there was increased probability of insulin vesicles in the region *r*_8_ due to the replenishment of RRP near the plasma membrane as secretion continued. Finally, at 150 min the probability of insulin vesicles in the region *r*_8_ was highest due to the completion of insulin RRP replenishment.

Moreover, we found in the regions *r*_2_ and *r*_3_ that the increasing probability from normal (0 min) to 15 min represents the maturation of immature vesicles, while the probability in *r*_7_ and *r*_8_ were decreased referring to the docked insulin vesicles release. Our results were consistent with previous studies [50, 51], confirming the validity of the analysis using the auto-segmentation method. Later at 30 and 150 min, newly matured insulin vesicles were transported, either to be secreted or to replenish the RRP [53], causing a decrease in probability at *r*_2_ and *r*_3_. Thus, regions *r*_2_—*r*_3_ were associated with new insulin vesicle maturation (Fig 3, pink region), regions *r*_4_—*r*_6_ to transportation (Fig 3, cyan region), and *r*_7_—*r*_8_ to secretion (Fig 3, violet region).

#### Hypothesis 1: Ex-4 boosts insulin secretion through the recruitment of mitochondria to meet the energy demand of insulin vesicle transportation

**Fig 4:**
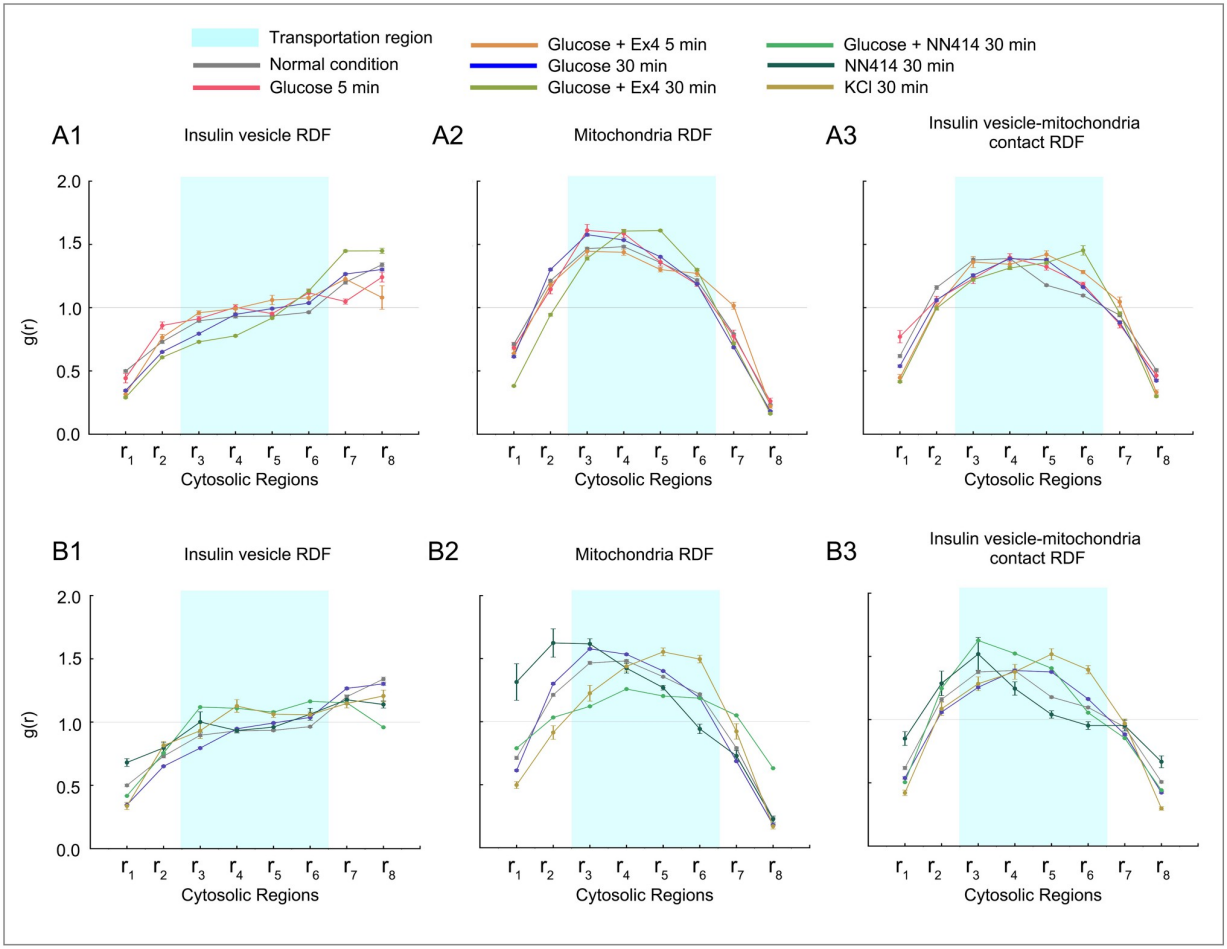
Radial distribution function (RDF) of insulin vesicle, mitochondria, and insulin vesicle-mitochondria contact under the various treatment conditions. (A1-A3) RDF distributions under glucose and Ex-4 treatment conditions compared to glucose treatment/normal condition. (B1-B3) RDF distributions under glucose and NN414 treatment conditions compared to glucose treatment/NN414 treatment/KCl treatment. Standard deviations are marked at each point to represent bias for datasets in the same condition.

We firstly compared distributions of insulin vesicles and mitochondria during treatments with glucose + Ex-4 (Fig 4A1–4A3). A lowered RDF in region *r*_8_ (closest to plasma membrane) is seen within 5 min of treatment with glucose + Ex-4 as compared to the treatment with glucose alone (Fig 4A1). Thus, an increased number of insulin vesicles were released from the RRP with the Ex-4 treatment. In the previous section, we also observed that the RRP was replenished at 30 min, we found that co-stimulation with Ex-4 enhanced this effect. Additionally, the RDF of vesicles at 30 min in transportation regions *r*_4_—*r*_6_ was lower with co-stimulation with Ex-4 compared to glucose alone and was higher in secretion regions *r*_7_—*r*_8_ (Fig 4A1). These observations indicated that Ex-4 facilitated the rapid movement of vesicles towards the plasma membrane.

Moreover, as can be seen from Fig 4A2, mitochondrial RDF in regions *r*_4_—*r*_6_ was relatively higher at 30 min treatment with Ex-4 as compared to the baseline and the treatment with glucose alone. It has been proposed that the distribution of mitochondria was to match energy demand inside of cells [54, 55]. Thus, such aggregation of mitochondria provides a local energy source to facilitate the movement of vesicles under glucose + Ex-4 treatment. Additionally, we observed higher probability of contacts between insulin vesicles and mitochondria in the transportation region when co-stimulated with Ex-4 (Fig 4A3). Thus, we hypothesize that Ex-4 boosts the insulin secretion by recruiting mitochondria to the transportation regions. Such recruitment give higher priority to satisfying the energy demand of insulin vesicle transportation towards the plasma membrane.

#### Hypothesis 2: Tifenazoxide (NN414) limits insulin secretion by reducing the energy supply for the maturation and transportation of insulin vesicles

As with Ex-4, we explored the effects of NN414 on insulin secretion by comparing insulin vesicle and mitochondria distributions during treatment with glucose + NN414 and NN414 alone (Fig 4B1–4B3). Since NN414 is a selective potassium channel opener and restricts insulin release, we also compared the NN414 condition with KCl treatment as KCl induces insulin secretion independent of glucose metabolism [56].

We found that at 30 min after treatment with NN414 alone, insulin vesicle probability was increased compared to baseline at regions *r*_2_ and *r*_3_ regions, which were related to insulin vesicle biosynthesis (Fig 4B1). After 30 min from treatment with glucose + NN414, we found that the RDF of insulin vesicle near the plasma membrane (regions *r*_7_—*r*_8_) was lower than the baseline and the treatment with glucose alone. Moreover, two significant differences in the RDF profiles under 30 min treatment with glucose + NN414 as compared to the 30 min treatments with only glucose and with glucose + Ex4: (I) the probability of mitochondria was lowered at maturation and transportation regions (*r*_2_ to *r*_6_) (Fig 4B2), (II) the probability of contacts between insulin vesicles and mitochondria were decreased at transportation region (Fig 4B3). These observations indicated that NN414 limits insulin secretion by detaining the recruitment of mitochondria and thus reducing the energy supply needed for the maturation and transportation of insulin vesicles (Fig 4B1).

In addition, we noticed that the mitochondria distribution changed drastically with NN414 treatment alone. Compared to cells with no treatment or with glucose + NN414 treatment, mitochondria in NN414-treated cells accumulated in the region close to the nucleus, as indicated by the increased probability in regions *r*_1_—*r*_3_ (Fig 4B2). Thus, we hypothesize that NN414 detain the recruitment of mitochondria regardless of glucose stimulation.

## Discussion

### A quick-and-clean approach to segment soft X-ray tomograms

In this paper, we presented an efficient and labor-saving auto-segmentation of tomograms using a combination of semantic segmentation and instance segmentation on different organelles as well as the analysis of the spatial distribution of segmented organelle labels using radial distribution function (RDF). Our pipeline was designed to overcome two drawbacks of conventional analysis. Firstly, manual segmentation was time consuming, generally taking 10–12 hours to segment a single INS-1E cell, which severely restricts SXT applications [57]. To address this issue, we conveyed deep learning frameworks for the auto-segmentation of all datasets, a process that takes only a couple of hours for training and tomogram segmentation. Secondly, the irregular cell shape is one of the major factors inducing bias to the final analysis. For example, Euclidean distances can describe the absolute distribution of insulin vesicles from the nucleus, but can hardly tell the difference between two vesicles within the same relative distance to the nucleus while one is in the squeezed region (S2C2 Fig, upper-right region) and one in the expanded region (S2C2 Fig, bottom-left region). To reduce this bias, we applied RDF to describe the distribution of insulin vesicles from the nucleus, which defined the organelle localization inside the cell regardless of the cell shape and normalizes the space in all directions. Our results allowed the definition of potential functional spaces within the cell where each type of organelle was involved in specific stages of the secretory pathway, highlighting probable shifts of the organelle distribution under different treatments and time of stimulation, and rationalized their hypothetical function at each stage.

To demonstrate the utility of this approach, we analyzed the insulin secretion process using soft X-ray tomography data for *β*-cells treated under different conditions and at multiple time points. We first compared the results with *White et al*. [3] to validate our pipeline. Then we extended our pipeline on new data to investigate the subcellular organelle rearrangement under new conditions. The consistence of our data increased the property and reliability of our analysis based on auto-segmentation on raw SXT tomograms. Although our method worked well in segmenting and exploring changes in cell architecture, it might be affected by the tomogram quality. As exemplified in the tomogram of cell ID 766_8, the LAC values on organelle voxels are low, leading to relatively low contrast ratio. This affects the segmentation accuracy, indicated by a low Recall index and decreased instance number (Tables 1–3). Since poor contrast reduces the accuracy of organelle detection, it will be important to develop an additional step for quality control during tomogram manual inspection to improve analysis and make the pipeline more robust. The quality of the segmented labels was expected to impact the subsequent analysis. For instance, low contrast ratio of mitochondria in SXT tomograms resulted in mislabeled voxels on both manual and auto- segmentation masks, indicated by the low Dice. This led to large deviations in the subsequent systematic analysis for LAC (Fig 2) and RDF (Fig 3). Further developments in image processing and segmentation methods will improve the quality of the segmented masks. Recently, a back-projection algorithm has been proposed to optimize details inside regions of interest to facilitate object recognition in image segmentation [58].

In addition, due to the similar contrast of several organelles in the cytosol, structures like microtubules and F-actin are hardly visible to soft X-ray beams. These obscures in SXT limit the identification of different organelles. Thus, we currently only focus on distinguishable organelles including insulin vesicles, mitochondria and nucleus to analyze their behaviors. This is expected to be improved by applying our method on tomograms obtained by correlative imaging combining SXT and fluorescence microscopy [59]. More importantly, despite our applications on *beta*-cells, this method also can be extended to other cell types, i.e., SXT tomograms of *Candida albicans* after treatments with antifungal peptoids [32].

### Biological implications

*White et al*. [3] discussed the impact of Ex-4 on insulin secretion: glucose and glucose + Ex-4 impact the insulin vesicle and mitochondria morphology during the secretion. We showed, in fact, despite the morphological variance (S6 Fig), Ex-4 also caused the aggregations of mitochondria in transportation regions glucose + Ex4 stimulation compared with glucose stimulation (Fig 4A). In the results, we also extended the same analysis of NN414 impact on *β*-cells. [60] pointed out that NN414 does not alter the mitochondrial oxidative metabolism and ATP production. As the observation of mitochondria behavior after glucose + NN414 stimulation, we hypothesized that NN414 affects the insulin secretion process by changing mitochondrial distribution without changing its function. This change in spatial organization of mitochondria further restricted the transportation and maturation of insulin vesicles. The results showed that as in the case of Ex-4, mitochondrial movement might be a prominent player in insulin vesicles maturation and secretion. However, the understanding of the exacted mechanism would need further investigation.

## Conclusion

In this paper, we present a novel auto-segmentation method based on deep learning techniques to segment SXT tomograms, followed by a systematic analysis pipeline. This approach generates high quality auto-segmentation masks of SXT images with improved accuracy and efficiency as compared to manual segmentation. To the best of our knowledge, it is the first time, we combine the semantic segmentation and instance segmentation on the same tomogram to obtain separate masks for individual organelles in a cell. Based on the systematic analysis, subcellular variances in different dynamic events are characterized in the mesoscale, including the organelle size, number, distributions. In addition, the systematic analysis based on RDF calculations provides a distinct perspective to characterize organelle distributions in whole-cells.

Our results provide insights into the *β*-cell structure and insulin vesicle dynamics during insulin secretion under glucose ± Ex-4 and NN414 treatments. Interestingly, both two potential drugs Ex-4 and NN414 are seen to play roles in insulin secretion by affecting the behavior of mitochondria: Ex-4 boosts insulin secretion through the recruitment of mitochondria to meet the energy demand of insulin vesicle transportation; Tifenazoxide (NN414) limits insulin secretion by reducing the energy supply for the maturation and transportation of insulin vesicles. Our method shows quantitative SXT tomogram processing to be a strong tool for subcellular structure recognition and drug discovery in whole-cell modeling effort.

## Supporting information

S1 Fig Organelle mask rendering and X-ray tomograms. (A) 3D rendering of manually-segmented organelle masks for the dataset 766_8. The render was performed by Amira version 6.7.0. (B) 2D orthoslice of a 3D X-ray tomogram from the front (B1), top (B2), and side (B3) views. (C) Organelle masks from a 2D orthoslice of the same dataset (front view).
(PDF)

S2 Fig Frames of semantic segmentation, instance segmentation and radial distribution function. (A) Overview of the U-Net framework used for semantic segmentation. Orange represents image features as they are processed through the 2D convolution, batch normalization (BN) and Rectified Linear Unit (ReLU). (B) Overview of the Mask R-CNN framework used for insulin vesicle instance segmentation. (C) Example showing eight cytosolic regions *r*_1_—*r*_8_ in a cell. These regions were used to compute the radial distribution function of different organelles.
(PDF)

S3 Fig Sketch of 3D fusion post-processing. An example voxel with red edges in the 3D image is segmented from all three axes. The final 3D label mask is constructed by merging 2D labels in each view. The example voxel is labeled along two axes (y and z, colored in gray) during semantic segmentation, and is thus labeled in the final 3D label mask.
(PDF)

S4 Fig Example of insulin vesicles labeled in manual segmentation but not in auto-segmentation. (A)Cropped 2D orthoslice of raw soft X-ray tomogram for Cell ID 766_8. Red and blue boxes show the organelle with an average LAC of 0.277 and 0.303, respectively. (B) Manually segmented mask labels two organelles as insulin vesicles. (C) Auto-segmented mask only labels the organelle with the LAC value of 0.303 as the insulin vesicle.
(PDF)

S5 Fig Example of insulin vesicles labeled in auto-segmentation but not in manual segmentation. (A) Cropped 2D orthoslice of raw soft X-ray tomogram for Cell ID 784_5. Red boxes show the region that contains no label in manually segmented mask but two labels in auto-segmented mask. (B) Manually segmented mask where two insulin vesicles are overlooked. (C) Auto-segmented mask showing correct prediction based on instance segmentation.
(PDF)

S6 Fig Comparison of organelles features and localization for all datasets under different conditions. Each feature is compared in one plots. Multi-comparison tests results are listed in S2 Table.
(PDF)

S7 Fig Dunnett significance test of the 5 treatment conditions. The significance tests were made on (A) insulin vesicle volume normalized by cytosol volume ratio, (B) insulin vesicle LAC value, (C) mitochondria volume normalized by cytosol volume, (D) mitochondria LAC value.
(PDF)

S1 Table Dice of the semantic segmentation before and after 3D fusion post-processing. (PDF)

S2 Table Treatment conditions for all cells. Cell numbers, average Linear absorption coefficient (LAC) values, and volumes of nucleus and cell masks are also listed.
(PDF)
