# Supplementary material for: Auto-segmentation and time-dependent systematic analysis of mesoscale cellular structure in β-cells during insulin secretion
Source: PLoS One. 2022 Mar 24;17(3):e0265567. doi: 10.1371/journal.pone.0265567 (PMC8947144; doi:10.1371/journal.pone.0265567)
Supplement: S1 Fig — (A) 3D rendering of manually-segmented organelle masks for the dataset 766_8. The render was performed by Amira version 6.7.0. (B) 2D orthoslice of a 3D X-ray tomogram from the front (B1), top (B2), and side (B3) views. (C) Organelle masks from a 2D orthoslice of the same dataset (front view). (PDF) [file pone.0265567.s001.pdf]

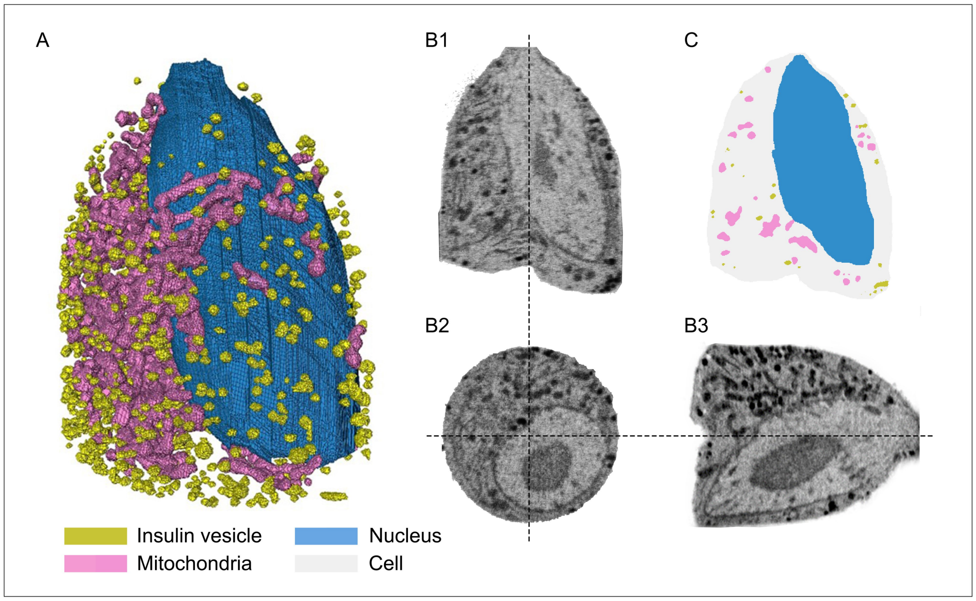

S1 Fig. Organelle mask rendering and X-ray tomograms. (A) 3D rendering of manually-segmented organelle masks for the dataset 766_8. The render was performed by Amira version 6.7.0. (B) 2D orthoslice of a 3D X-ray tomogram from the front (B1), top (B2), and side (B3) views. (C) Organelle masks from a 2D orthoslice of the same dataset, (front view).
