# Supplementary material for: Auto-segmentation and time-dependent systematic analysis of mesoscale cellular structure in β-cells during insulin secretion
Source: PLoS One. 2022 Mar 24;17(3):e0265567. doi: 10.1371/journal.pone.0265567 (PMC8947144; doi:10.1371/journal.pone.0265567)
Supplement: S2 Fig — (A) Overview of the U-Net framework used for semantic segmentation. Orange represents image features as they are processed through the 2D convolution, batch normalization (BN) and Rectified Linear Unit (ReLU). (B) Overview of the Mask R-CNN framework used for insulin vesicle instance segmentation. (C) Example showing eight cytosolic regions r1—r8 in a cell. These regions were used to compute the radial distribution function of different organelles. (PDF) [file pone.0265567.s002.pdf]

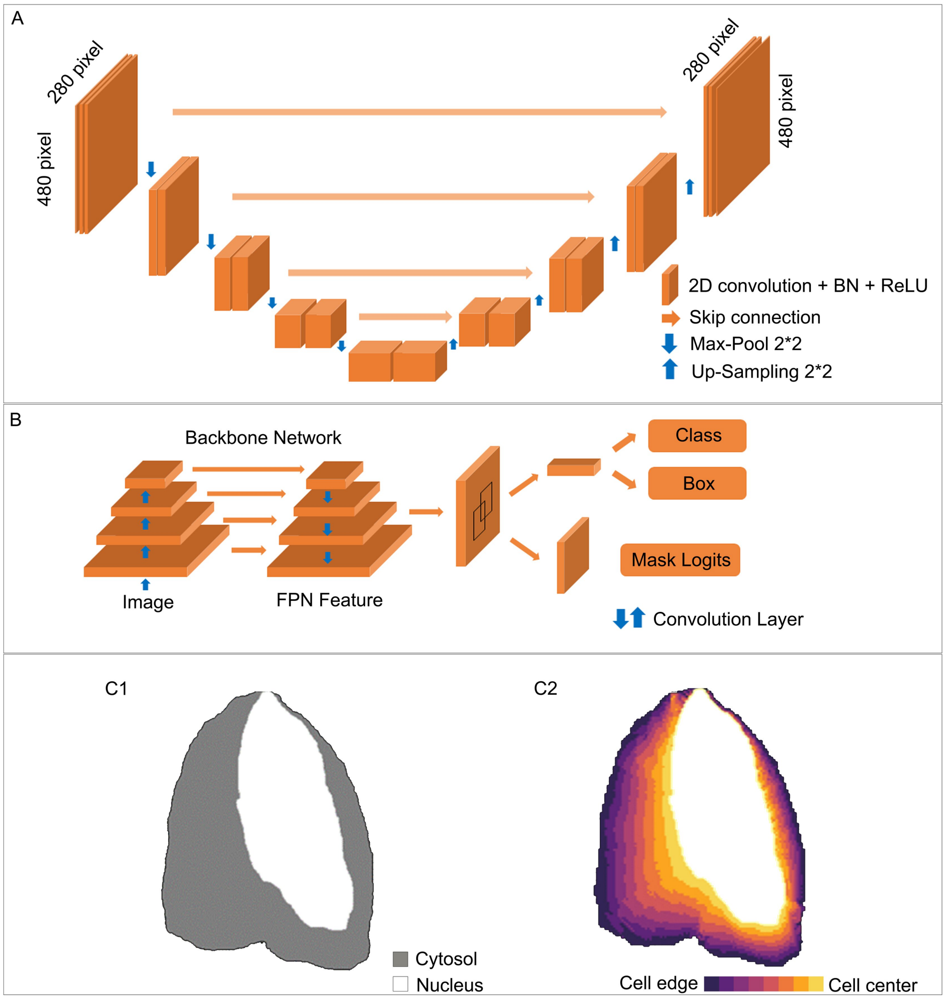

S2 Fig. Frames of semantic segmentation, instance segmentation and radial distribution function. (A) Overview of the U-Net framework used for semantic segmentation. Orange represents image features as they are processed through the 2D convolution, batch normalization (BN) and Rectified Linear Unit (ReLU). (B) Overview of the Mask R-CNN framework used for insulin vesicle instance segmentation. (C) Example showing eight cytosolic regions r_1 - r_8 in a cell. These regions were used to compute the radial distribution function of different organelles.
