# Supplementary material for: Auto-segmentation and time-dependent systematic analysis of mesoscale cellular structure in β-cells during insulin secretion
Source: PLoS One. 2022 Mar 24;17(3):e0265567. doi: 10.1371/journal.pone.0265567 (PMC8947144; doi:10.1371/journal.pone.0265567)
Supplement: S3 Fig — An example voxel with red edges in the 3D image is segmented from all three axes. The final 3D label mask is constructed by merging 2D labels in each view. The example voxel is labeled along two axes (y and z, colored in gray) during semantic segmentation, and is thus labeled in the final 3D label mask. (PDF) [file pone.0265567.s003.pdf]

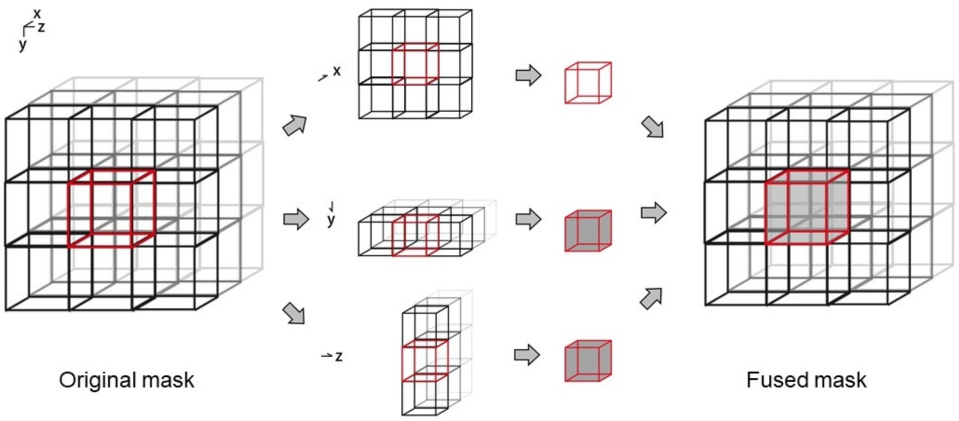

S3 Fig. Sketch of 3D fusion post-processing. An example voxel with red edges in the 3D image is segmented from all three axes. The final 3D label mask is constructed by merging 2D labels in each view. The example voxel is labeled along two axes (y and z, colored in gray) during semantic segmentation, and is thus labeled in the final 3D label mask.
