# Supplementary material for: Auto-segmentation and time-dependent systematic analysis of mesoscale cellular structure in β-cells during insulin secretion
Source: PLoS One. 2022 Mar 24;17(3):e0265567. doi: 10.1371/journal.pone.0265567 (PMC8947144; doi:10.1371/journal.pone.0265567)
Supplement: S4 Fig — (A)Cropped 2D orthoslice of raw soft X-ray tomogram for Cell ID 766_8. Red and blue boxes show the organelle with an average LAC of 0.277 and 0.303, respectively. (B) Manually segmented mask labels two organelles as insulin vesicles. (C) Auto-segmented mask only labels the organelle with the LAC value of 0.303 as the insulin vesicle. (PDF) [file pone.0265567.s004.pdf]

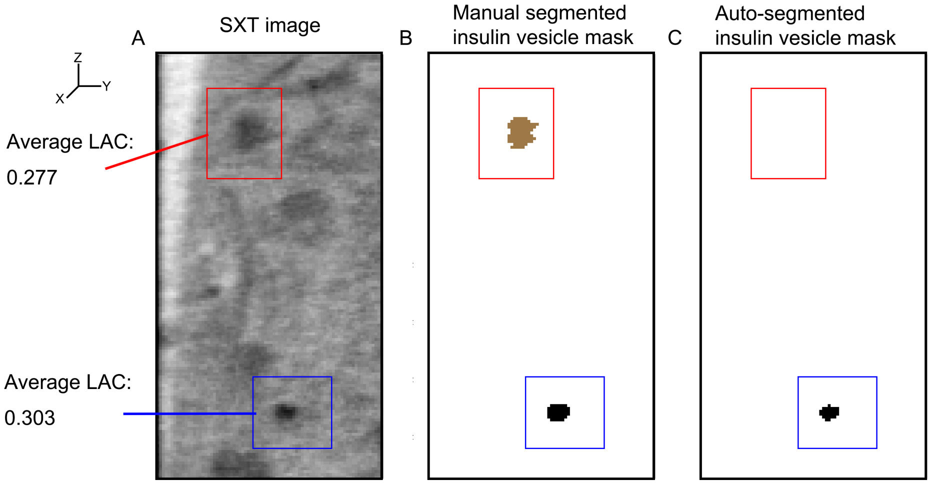

S4 Fig. Example of insulin vesicles labeled in manual segmentation but not in auto-segmentation. (A)Cropped 2D orthoslice of raw soft X-ray tomogram for Cell ID 766_8. Red and blue boxes show the organelle with an average LAC of 0.277 and 0.303, respectively. (B) Manually segmented mask labels two organelles as insulin vesicles. (C) Auto-segmented mask only labels the organelle with the LAC value of 0.303 as the insulin vesicle.
