# Supplementary material for: Auto-segmentation and time-dependent systematic analysis of mesoscale cellular structure in β-cells during insulin secretion
Source: PLoS One. 2022 Mar 24;17(3):e0265567. doi: 10.1371/journal.pone.0265567 (PMC8947144; doi:10.1371/journal.pone.0265567)
Supplement: S5 Fig — (A) Cropped 2D orthoslice of raw soft X-ray tomogram for Cell ID 784_5. Red boxes show the region that contains no label in manually segmented mask but two labels in auto-segmented mask. (B) Manually segmented mask where two insulin vesicles are overlooked. (C) Auto-segmented mask showing correct prediction based on instance segmentation. (PDF) [file pone.0265567.s005.pdf]

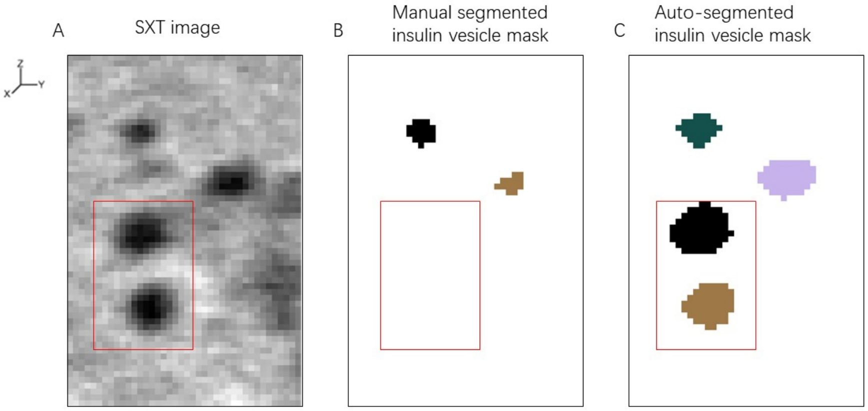

S5 Fig. Example of insulin vesicles labeled in auto-segmentation but not in manual segmentation. (A) Cropped 2D orthoslice of raw soft X-ray tomogram for Cell ID 784_5. Red boxes show the region that contains no label in manually segmented mask but two labels in auto-segmented mask. (B) Manually segmented mask where two insulin vesicles are overlooked. (C) Auto-segmented mask showing correct prediction based on instance segmentation.
