# Supplementary material for: Auto-segmentation and time-dependent systematic analysis of mesoscale cellular structure in β-cells during insulin secretion
Source: PLoS One. 2022 Mar 24;17(3):e0265567. doi: 10.1371/journal.pone.0265567 (PMC8947144; doi:10.1371/journal.pone.0265567)
Supplement: S6 Fig — Each feature is compared in one plots. Multi-comparison tests results are listed in S2 Table. (PDF) [file pone.0265567.s006.pdf]

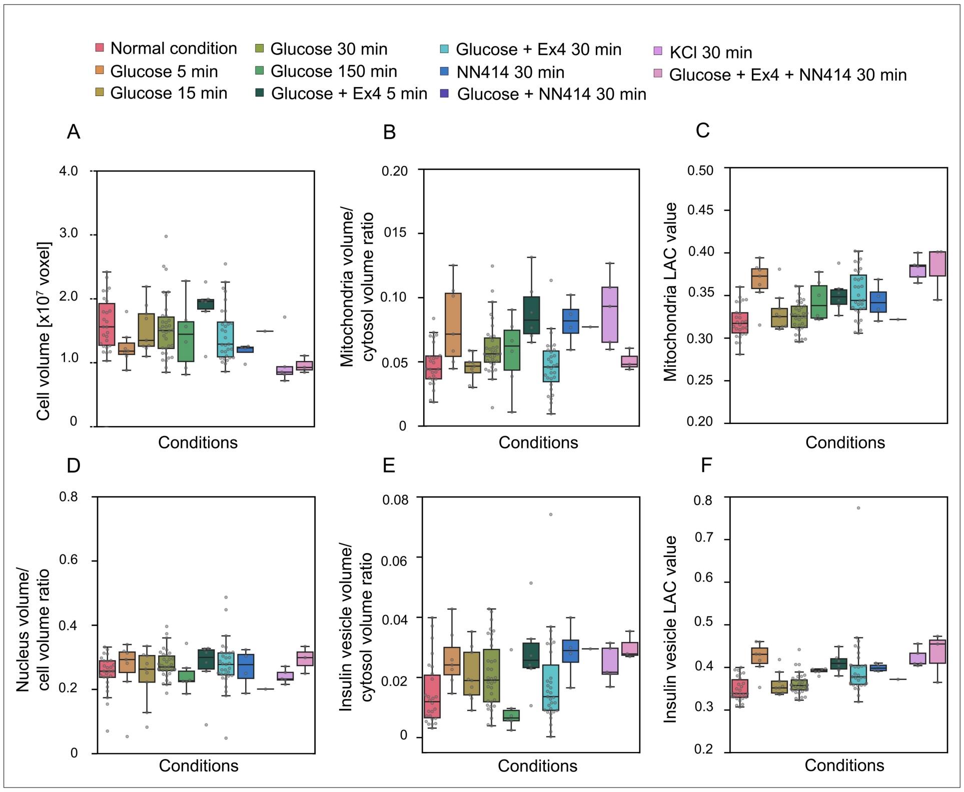

S6 Fig. Comparison of organelles features and localization for all datasets under different conditions. Each feature is compared in one plots. Multi-comparison tests results are listed in S1 Table.
