# Supplementary material for: Auto-segmentation and time-dependent systematic analysis of mesoscale cellular structure in β-cells during insulin secretion
Source: PLoS One. 2022 Mar 24;17(3):e0265567. doi: 10.1371/journal.pone.0265567 (PMC8947144; doi:10.1371/journal.pone.0265567)
Supplement: S7 Fig — The significance tests were made on (A) insulin vesicle volume normalized by cytosol volume ratio, (B) insulin vesicle LAC value, (C) mitochondria volume normalized by cytosol volume, (D) mitochondria LAC value. (PDF) [file pone.0265567.s007.pdf]

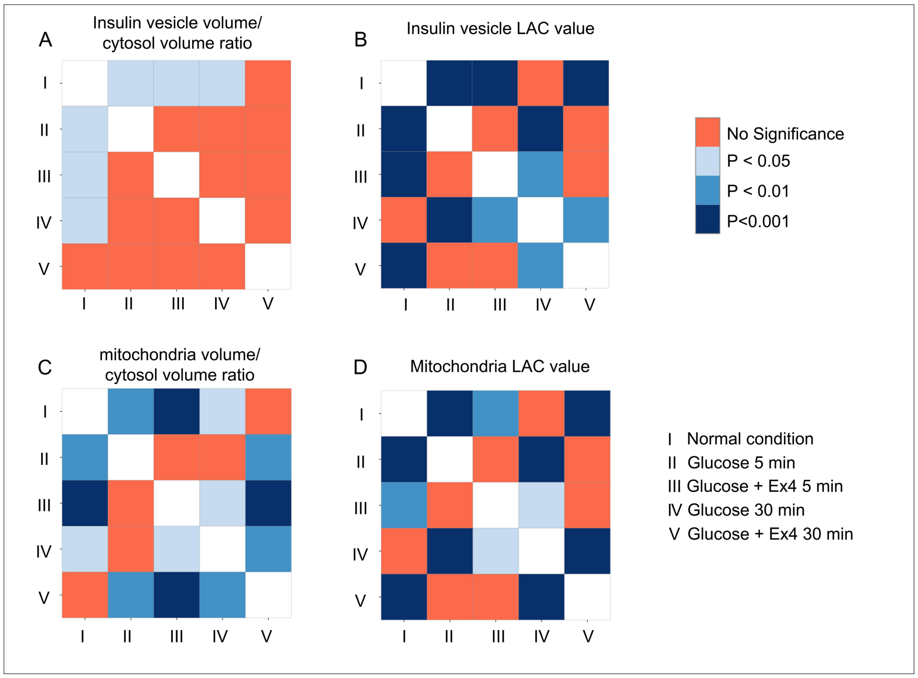

S7 Fig. Dunnett significance test of the 5 treatment conditions. The significance tests were made on (A) insulin vesicle volume normalized by cytosol volume ratio, (B) insulin vesicle LAC value, (C) mitochondria volume normalized by cytosol volume, (D) mitochondria LAC value.
