# Supplementary material for: Auto-segmentation and time-dependent systematic analysis of mesoscale cellular structure in β-cells during insulin secretion
Source: PLoS One. 2022 Mar 24;17(3):e0265567. doi: 10.1371/journal.pone.0265567 (PMC8947144; doi:10.1371/journal.pone.0265567)
Supplement: S1 Table — (PDF) [file pone.0265567.s008.pdf]

S1 Table Dice of the semantic segmentation before and after 3D fusion post-processing.

Dataset	Cell		Nucleus		Mitochondria	
	Before (%)	After (%)	Before (%)	After (%)	Before (%)	After (%)
766_8	90.74	93.22	93.21	93.88	68.58	70.34
784_5	87.43	87.83	89.95	91.82	63.17	67.29
842_17	85.34	92.15	93.50	89.49	65.03	67.40
Average	87.84	91.60	88.89	91.74	65.59	68.34
