# Supplementary material for: Auto-segmentation and time-dependent systematic analysis of mesoscale cellular structure in β-cells during insulin secretion
Source: PLoS One. 2022 Mar 24;17(3):e0265567. doi: 10.1371/journal.pone.0265567 (PMC8947144; doi:10.1371/journal.pone.0265567)
Supplement: S2 Table — Cell numbers, average Linear absorption coefficient (LAC) values, and volumes of nucleus and cell masks are also listed. (PDF) [file pone.0265567.s009.pdf]

S2 Table Treatment conditions for all cells.

Conditions			Number of Datasets	Cell volume		Nucleus volume		nucleus size (nucleus volume/ Cell volume ,%)	Cell LAC		Nucleus LAC		Mitochondria LAC		Insulin vesicle LAC		Insulin vesicle number	
Treatment	Glucose concentration (mM)	Treatment time (min)		Mean (voxel num.)	SE(voxel number)	Mean (voxel num.)	SE (voxel number)		Mean (μm^{-1})	SE (μm^{-1})	Mean (μm^{-1})	SE (μm^{-1})	Mean (μm^{-1})	SE (μm^{-1})	Mean (μm^{-1})	SE (μm^{-1})	Number mean	Number SE
Normal	/	/	26	1.50E+07	6.94E+05	3.86E+06	1.64E+05	25.79%	2.49E-01	2.62E-03	2.42E-01	2.37E-03	3.16E-01	3.77E-03	3.44E-01	5.34E-03	514	47
Glucose	25	5	7	1.25E+07	1.09E+06	3.32E+06	4.91E+05	26.57%	2.98E-01	1.11E-02	2.86E-01	7.84E-03	3.66E-01	9.81E-03	4.22E-01	1.40E-02	791	123
Glucose	25	15	9	1.53E+07	1.24E+06	3.64E+06	4.27E+05	23.83%	2.57E-01	1.01E-02	2.48E-01	7.13E-03	3.30E-01	7.57E-03	3.61E-01	9.00E-03	862	84
Glucose	25	30	34	1.54E+07	8.79E+05	4.25E+06	2.25E+05	27.60%	2.55E-01	3.75E-03	2.43E-01	3.64E-03	3.24E-01	3.05E-03	3.62E-01	4.44E-03	780	68
Glucose	25	150	6	1.43E+07	2.19E+06	3.44E+06	5.73E+05	24.13%	2.81E-01	7.27E-03	2.72E-01	6.55E-03	3.44E-01	9.78E-03	3.91E-01	2.62E-03	463	175
Glucose + Ex-4	25	5	6	1.85E+07	1.62E+06	4.89E+06	7.91E+05	26.49%	2.84E-01	5.93E-03	2.69E-01	6.34E-03	3.51E-01	8.61E-03	4.10E-01	9.79E-03	1071	174
Glucose + Ex-4	25	30	31	1.45E+07	7.67E+05	4.06E+06	3.43E+05	27.94%	2.75E-01	4.35E-03	2.59E-01	5.06E-03	3.52E-01	5.11E-03	4.00E-01	1.41E-02	619	61
Glucose + Ex-4 + NN414	25	30	3	9.62E+06	7.71E+05	2.85E+06	4.31E+05	29.63%	3.07E-01	1.73E-02	2.96E-01	1.50E-02	3.82E-01	1.88E-02	4.31E-01	3.35E-02	759	75
Glucose + NN414	25	30	1	1.49E+07	/	3.00E+06	/	20.12%	2.47E-01	/	2.46E-01	/	3.22E-01	/	3.72E-01	/	798	/
Normal + NN414	0	30	4	1.16E+07	9.05E+05	3.22E+06	7.00E+05	27.83%	2.80E-01	1.23E-02	2.78E-01	8.72E-03	3.51E-01	1.01E-02	4.01E-01	5.45E-03	669	90
KCl	0	30	5	1.01E+07	1.80E+06	2.41E+06	4.10E+05	23.96%	3.14E-01	5.54E-03	3.04E-01	6.99E-03	3.81E-01	6.13E-03	4.22E-01	9.82E-03	559	89

Cell numbers, average LAC values, and volumes of nucleus and cell masks are also listed.
